# RvD1 accelerates the resolution of inflammation by promoting apoptosis of the recruited macrophages via the ALX/FasL-FasR/caspase-3 signaling pathway

**DOI:** 10.1038/s41420-021-00708-5

**Published:** 2021-11-08

**Authors:** Shu-yang Xiang, Yang Ye, Qian Yang, Hao- ran Xu, Chen-xi Shen, Min-qi Ma, Shao-wu Jin, Hong-xia Mei, Sheng-xing Zheng, Fang-gao Smith, Sheng-wei Jin, Qian Wang

**Affiliations:** 1 Department of Anesthesia and Critical Care, The Second Affiliated Hospital and Yuying Children’s Hospital of Wenzhou Medical University, Zhejiang, 325027 China; 2 nstitute of Inflammation and Aging, College of Medical and Dental Sciences, University of Birmingham, Birmingham, UK

**Keywords:** Acute inflammation, Respiratory distress syndrome

## Abstract

The uncontrolled inflammatory response caused by a disorder in inflammation resolution is one of the reasons for acute respiratory distress syndrome (ARDS). The macrophage pool markedly expands when inflammatory monocytes, known as recruited macrophages, migrate from the circulation to the lung. The persistent presence of recruited macrophages leads to chronic inflammation in the resolution phase of inflammation. On the contrary, elimination of the recruited macrophages at the injury site leads to the rapid resolution of inflammation. Resolvin D1 (RvD1) is an endogenous lipid mediator derived from docosahexaenoic acid. Mice were administered RvD1 via the tail vein 3 and 4 days after stimulation with lipopolysaccharide. RvD1 reduced the levels of the inflammatory factors in the lung tissue, promoted the anti-inflammatory M2 phenotype, and enhanced the phagocytic function of recruited macrophages to alleviate acute lung injury. We also found that the number of macrophages was decreased in BAL fluid after treatment with RvD1. RvD1 increased the apoptosis of recruited macrophages partly via the FasL-FasR/caspase-3 signaling pathway, and this effect could be blocked by Boc-2, an ALX/PRP2 inhibitor. Taken together, our findings reinforce the concept of therapeutic targeting leading to the apoptosis of recruited macrophages. Thus, RvD1 may provide a new therapy for the resolution of ARDS.

## Introduction

Acute respiratory distress syndrome (ARDS) is a common critical condition that results in high mortality [1]. Its pathophysiological mechanism manifests as a severe inflammatory response that is characterized by the infiltration of large inflammatory cells and cytokine production [2]. Recruited macrophages, derived from the mononuclear cells of the bone marrow, accumulate in the lungs following an injury [3–5]. The persistent presence of recruited macrophages at the injury site, which mainly constitutes the pro-inflammatory M1 phenotype, leads to chronic inflammation [5]. Therefore, removal of the excessive recruited macrophages is important to expedite the alleviation of inflammation and protect the lung tissue.

Several molecules that play a role in alleviating inflammation have been elucidated and named as special anti-inflammatory mediators (SPM). These molecules exert anti-inflammatory and anti-infective effects [6]. Resolvin D1 (RvD1) is an endogenous lipid mediator derived from docosahexaenoic acid (DHA), which has been proven to exert anti-inflammatory effects in several disease models [7]. RvD1 limits the infiltration of neutrophils during the subsided phase of inflammation, regulates the migration of human polymorphonuclear leukocyte-endothelial cells [8], inhibits the release of pro-inflammatory cytokines [9], and enhances phagocytic function and cell apoptosis [10]. During acute lung injury, macrophages are divided into resident and recruiting macrophages [11]. Resident macrophages have a certain degree of self-maintenance in a stable state [12] and are the key coordinators of inflammation and repair in the lungs [13]. The recruitment of macrophages stems from monocytes circulating in the blood. Monocyte chemotactic protein (MCP)-1 promotes the recruitment of monocytes to inflammatory tissues to perform their functions [14]. These two cell types play different functions in acute lung injury. Studies show that the depletion and recruitment of macrophages can reduce acute allergic lung inflammation [15]. Elimination of macrophages from chronic injury sites results in the rapid alleviation of inflammation [16, 17]. Additionally, the recruited macrophages undergo apoptosis during inflammation [18] and are subsequently cleared by the neighboring phagocytes, which also constitutes a necessary step in the resolution of acute lung inflammation [19].

In this study, we determined the effect of RvD1 on the recruitment of macrophages during the resolution phase of lipopolysaccharide (LPS)-induced inflammation. We found that RvD1 administered at the peak of inflammation enhanced efferocytosis and M2 phenotype in the recruited macrophages and reduced the production of inflammatory cytokines. Moreover, RvD1 could remove the recruited macrophages by accelerating ALX/Fas ligand (FasL)-FasR/caspase-3-mediated apoptosis and rapidly promoted the resolution of inflammation.

## Results

### RvD1 attenuates LPS-induced lung injury to promote resolution of inflammation

**Fig. 1:**
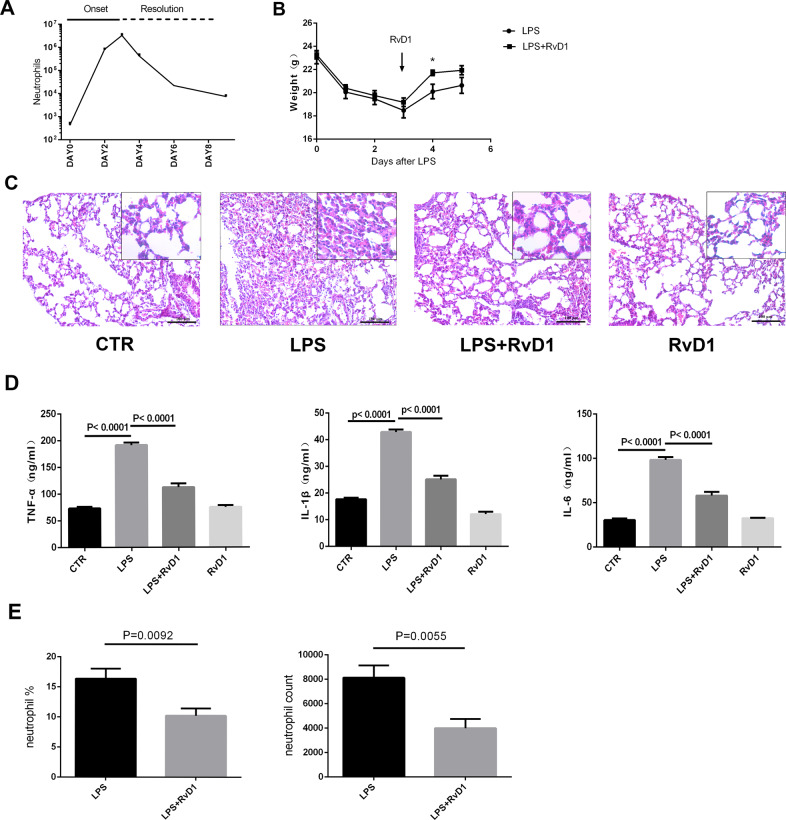
RvD1 attenuates LPS-induced lung injury to promote resolution of inflammation. **A** After LPS stimulation, neutrophil counts in the BALF were detected using flow cytometry. **B** RvD1 was administered through the tail vein on day 3 and the weights of mice were determined. **C** Treatment with RvD1 protected the lung tissues in LPS-induced ARDS. **D** Aerosol inhalation of LPS significantly increased TNF-α, IL-1β, and IL-6 levels in lung tissue, and RvD1 treatment decreased the levels of these inflammatory factors. **E** Treatment with RvD1 reduced infiltration of neutrophils in the BALF.

After the intratracheal administration of LPS (1 mg/kg), the number of neutrophils in the BALF increased sharply, peaked on the third day, and then decreased to a steady state by day 8 (Fig. 1A). The body weights of mice showed a decreasing trend and reached the minimum on the third day (Fig. 1B). Therefore, the third day was the peak of inflammation, and after this day, a resolution phase was observed. Mice were administered RvD1 on the third day. After 24 h, the weight of mice in the LPS + RvD1 group increased compared to those in the LPS group (*P* < 0.05) (Fig. 1B). Moreover, the general condition of mice improved, and the appetite of mice in the RvD1 treatment group increased.

The lung tissues of mice in the LPS group were seriously damaged. RvD1 treatment alleviated lung tissue damage (Fig. 1C). The lung injury score was significantly reduced (S1). Furthermore, levels of the inflammatory factors TNF-α, IL-1β, and IL-6 in the lung tissue homogenate were decreased in mice in the LPS + RvD1 group compared with those in the LPS group (Fig. 1D). However, there were no significant differences in the levels of these markers between the CTR and RvD1 groups.

The number of neutrophils decreased and was one of the important indicators of the resolution of inflammation. On the fifth day, the F4/80-LY6C + neutrophils in the BALF decreased in the LPS + RvD1 group compared with the LPS group (*P* < 0.05) (Fig. 1E).

### RvD1 reduces levels of the recruited macrophages in the resolution phase

**Fig. 2:**
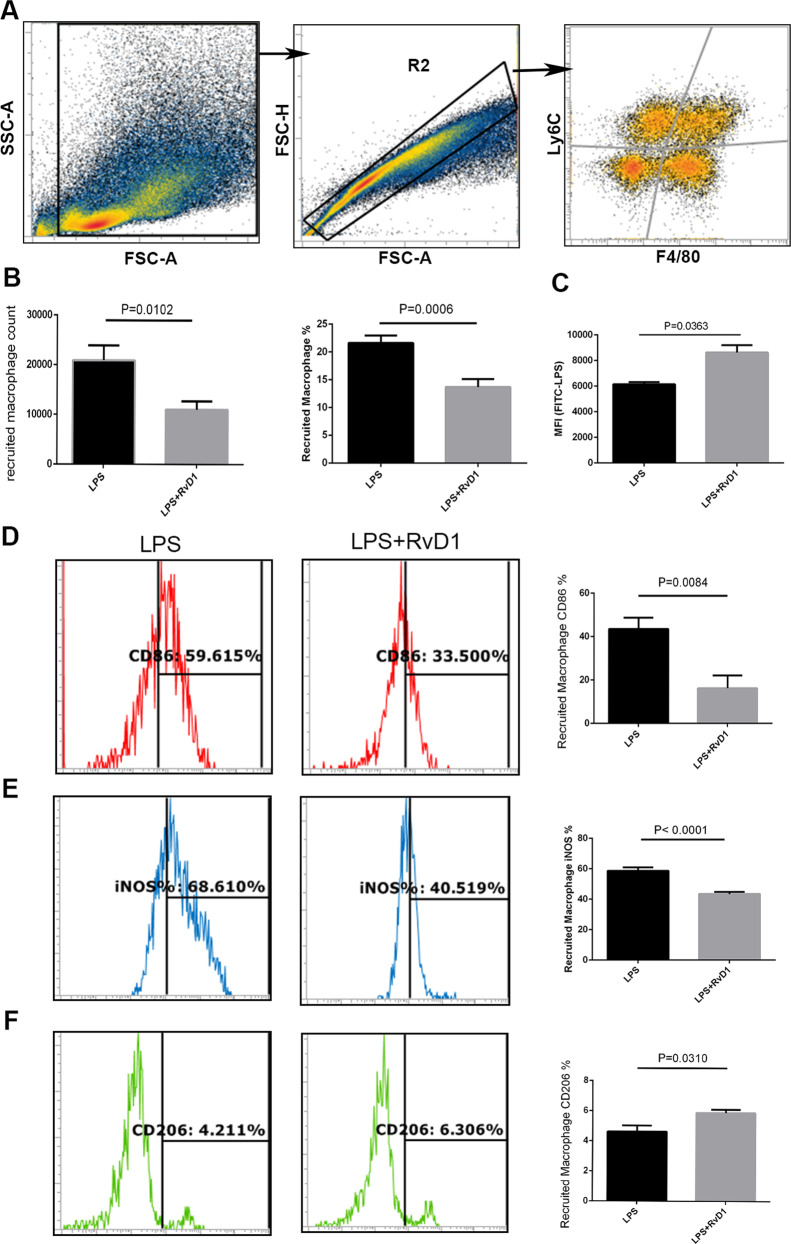
RvD1 upregulates the recruited macrophages M2 phenotype, enhances phagocytic function, and reduces their accumulation in vivo. **A** Distinguished the recruited macrophages with F4/80 + LY6C +. **B** Treatment with RvD1 reduced the number of recruited macrophages in the BALF. **C** Determination of fluorescence intensity of LPS in the recruited macrophages from BALF after 12 h. Expression of CD86 (**D**), iNOS (**E**), and CD206 (**F**) in BALF from mice were measured in recruited macrophages in LPS with or without RvD1 administration.

The recruited macrophages were identified as F4/80 + LY6C + (Fig. 2A). The number and percentage of recruited macrophages in the alveolar lavage fluid decreased in the LPS + RvD1 group compared with the LPS group (Fig. 2B). The phagocytosis of the recruited macrophages was determined. The mean fluorescence intensity (MFI) of FITC-LPS on the recruited macrophages in the RvD1 treatment group was higher than that in the LPS group (Fig. 2C). Moreover, treatment with RvD1 reduced the expression of CD86 and iNOS levels (Fig. 2F) and increased CD206 levels in the recruited macrophages (Fig. 2G).

### RvD1 increases the numbers of the recruited macrophages in the lymph nodes

**Fig. 3:**
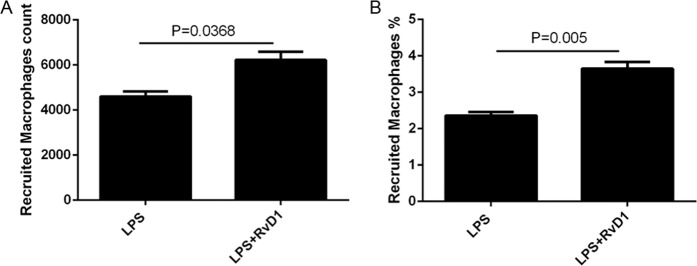
RvD1 increases the numbers of recruited macrophages in the lymph nodes. The numbers (**A**) and percentage (**B**) of the recruited macrophages in the pulmonary lymph node homogenate were detected using flow cytometry.

Next, we attempted to elucidate why the levels of the recruited macrophages decreased after treatment with RvD1. The pulmonary lymph node homogenate was used to assess the number of recruited macrophages using flow cytometry. As shown in Fig. 3A, B, the number and percentage of the recruited macrophages in the homogenate were increased in the LPS + RvD1 group compared with the LPS group.

### RvD1 promotes apoptosis of recruited macrophages in LPS-induced lung injury in vivo

**Fig. 4:**
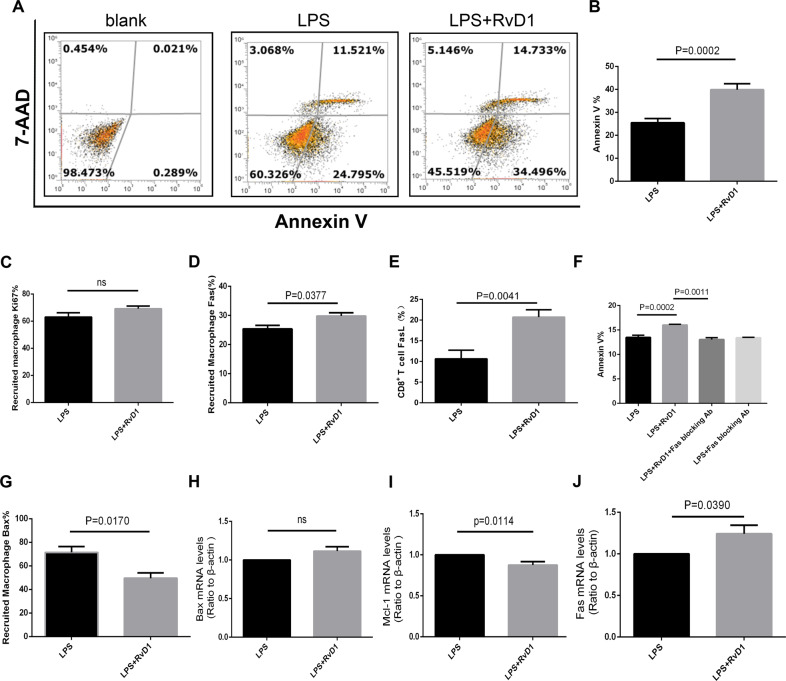
RvD1 promotes apoptosis of recruited macrophages in LPS-induced lung injury in vivo. **A** Staining clusters of Annexin V and 7-AAD on recruited macrophages. **B** Percentage of cells expressing Annexin V^+^7-AAD^−^ on recruited macrophages. **C** Effect of RvD1 on the proliferation of recruited macrophages. **D** Fas levels (%) on the recruited macrophages. **E** FasL levels (%) on CD8^+ ^T cells. **F** The effect of Fas-blocking antibody on the apoptosis of recruited macrophage. (G) Bax expression (%) on recruited macrophages. Expression of Bax (**H**), Mcl-2 (**I**), Fas (**J**) mRNA in the recruited macrophages.

Next, the apoptosis and proliferation in the recruited macrophages were measured using flow cytometry. As shown in Fig. 4A–C, treatment with RvD1 promoted apoptosis of the recruited macrophages; however, there were no effects on proliferation.

Levels of the apoptosis-related proteins were determined using flow cytometry and RT-PCR. Levels of the pro-apoptosis protein, Fas, were increased in the LPS + RvD1 group compared with the LPS group in the recruited macrophages (*p* = 0.0377) (Fig. 4D). The FasL in CD8^+^ T cells was much higher in the LPS + RvD1 group compared with the LPS group (*p* = 0.0041) (Fig. 4E). Treatment with Fas-blocking antibody (LPS + RvD1+FasL blocking Ab group) reduced the cell apoptosis compared with the LPS + RvD1 group (*p* = 0.0011) (Fig. 4F). Bax was decreased in the LPS + RvD1 group compared with the LPS group in the recruited macrophages (*p* = 0.0170) (Fig. 4G). To further elucidate the mechanism of macrophage apoptosis, the recruited macrophages were sorted to extract RNA. Fas expression was increased, Bax gene expression was unchanged, and intracellular Mcl-1 gene expression was decreased in the LPS + RvD1 group compared to that in the LPS group (Fig. 4H–J).

### Protective effects of RvD1 on LPS-induced BMDMs in vitro

**Fig. 5:**
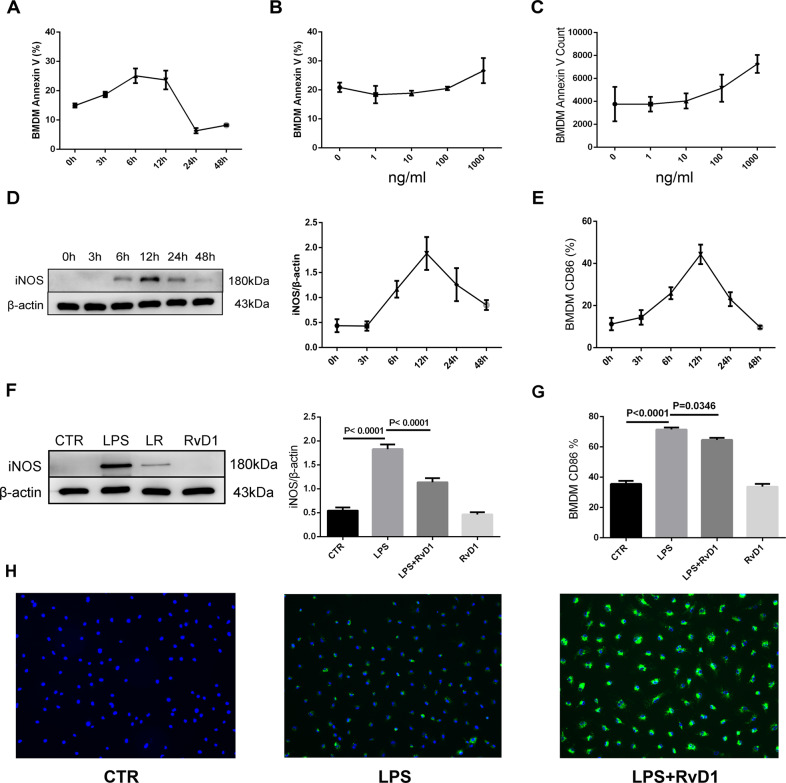
Protective effects of RvD1 on LPS-induced BMDMs in vitro. **A** Time-course of LPS-induced apoptosis. About 10^6^ macrophages were treated with 1 μg/mL LPS at the indicated time. B) Cells were treated for 12 h with the indicated. concentrations of LPS; **C** Annexin V was detected as indicated above. **D**. Expression of LPS-induced iNOS was measured using western blotting. **E** LPS induces the expression of CD86. **F** Western blotting was used to determine the expression of iNOS in BMDMs treated with 1 μg/mL of LPS in the presence or absence of 100 nM RvD1. **G** RvD1 inhibits LPS-induced CD86 expression. **H** Macrophages were treated with 1 μg/mL FITC-labeled LPS (green) for 12 h and intracellular fluorescence was analyzed using fluorescence microscopy. DAPI nuclear staining was used to label the macrophage (blue), efferocytosis was observed via the number of LPS (green) containing in macrophages (blue).

LPS induced the apoptosis of macrophages in a time- and dose-dependent manner. Treatment with 1 μg/mL of LPS resulted in the apoptosis of macrophages, which peaked at 6–12 h (Fig. 5A). Different concentrations of LPS, including 1, 10, 100, and 1000 ng/mL, were used in this study. We found that 1000 ng/mL LPS led to the maximum level of apoptosis of the macrophages, and this concentration was used in subsequent experiments (Fig. 5B, C).

iNOS levels were detected using western blotting, and the protein expression of iNOS reached its peak at 12 h and then decreased (Fig. 5D). At the same time, CD86 levels also peaked at 12 h (Fig. 5E). Therefore, in this study, cells were stimulated with LPS for 12 h in the presence or absence of RvD1.

As shown in Fig. 5F, G, the increased expression of iNOS and CD86 was abolished by treatment with RvD1. Moreover, treatment with RvD1 also promoted the phagocytosis of fluorescent LPS by the macrophages (Fig. 5H).

### RvD1 promotes apoptosis of BMDMs through Fas

**Fig. 6:**
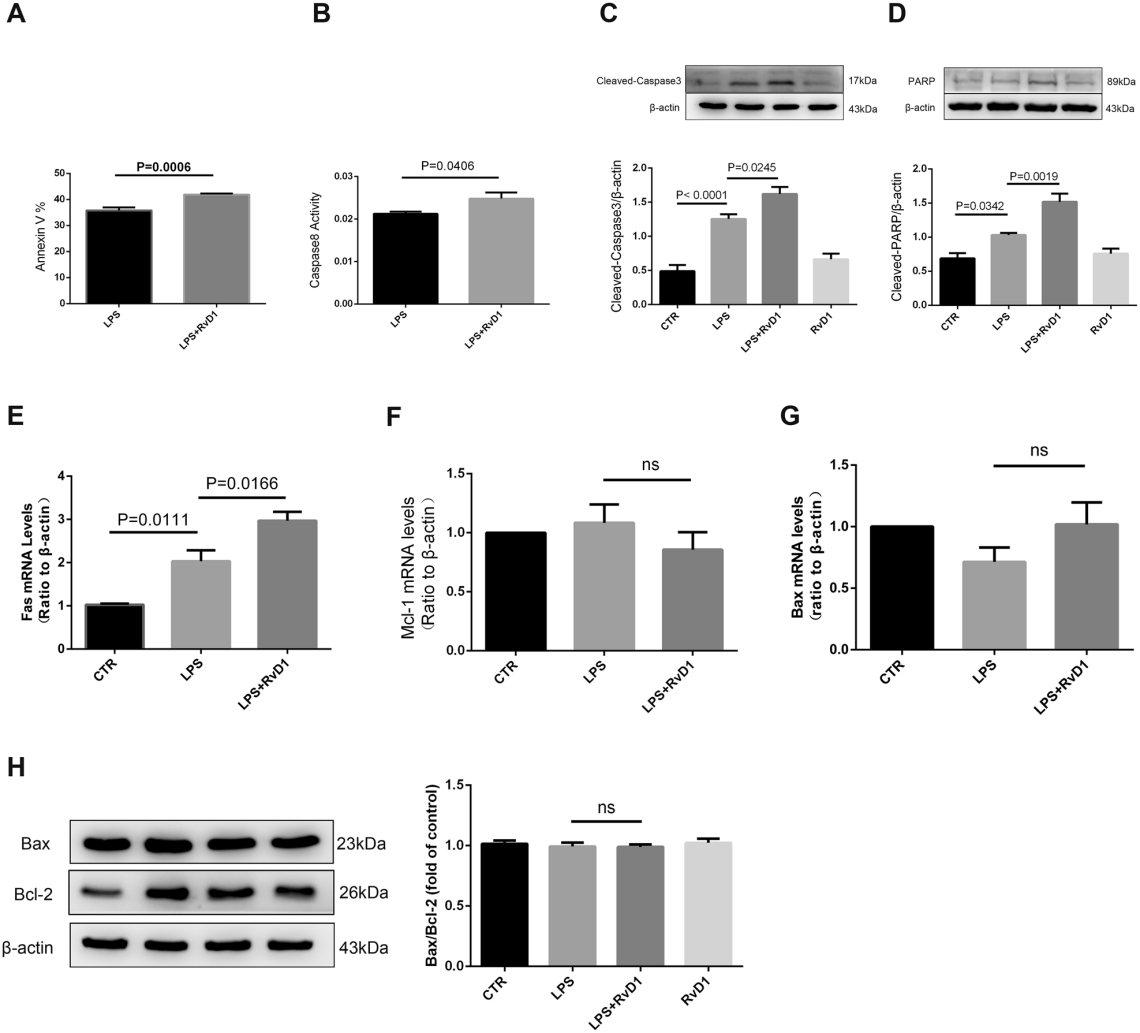
RvD1 promotes apoptosis of BMDMs through Fas. **A** RvD1 promotes LPS-induced apoptosis in BMDMs. **B** Caspase 8 activity was also measured in BMDMs. Expression of cleaved caspase-3 (**C**) and cleaved PARP (**D**) was measured using western blotting. mRNA expression of Fas (**E**), Mcl-1 (**F**), and Bax (**G**) was measured using qPCR. **H** Western blot was used to confirm that RvD1 has no effect on Bax/Bcl-2.

The primary cells were cultured with LPS and/or RvD1 for 12 h and the extent of apoptosis was determined using flow cytometry. As shown in Fig. 6A, treatment with RvD1 enhanced apoptosis (*p* = 0.0006). Compared to that in the LPS group, the caspase 8 activity was increased in the LPS + RvD1 group (*p* = 0.0406). Compared to that in the CTR group, the protein expression of cleaved caspase-3 and cleaved PARP was increased in the LPS group. This increase in protein expression was abrogated by treatment with RvD1 (Fig. 6C, D). Meanwhile, the mRNA expression of Fas was increased in the LPS + RvD1 group compared with the LPS group (Fig. 6E). However, there were no significant differences in the mRNA expression of Bax and Mcl-1 between the LPS and LPS + RvD1 groups (Fig. 6F, G). Moreover, there were no changes in the level of Bax/Bcl-2 protein between the LPS and LPS + RvD1 groups (Fig. 6H).

### The protective effect of RvD1 on lung tissues is dependent on ALX/PRP2

**Fig. 7:**
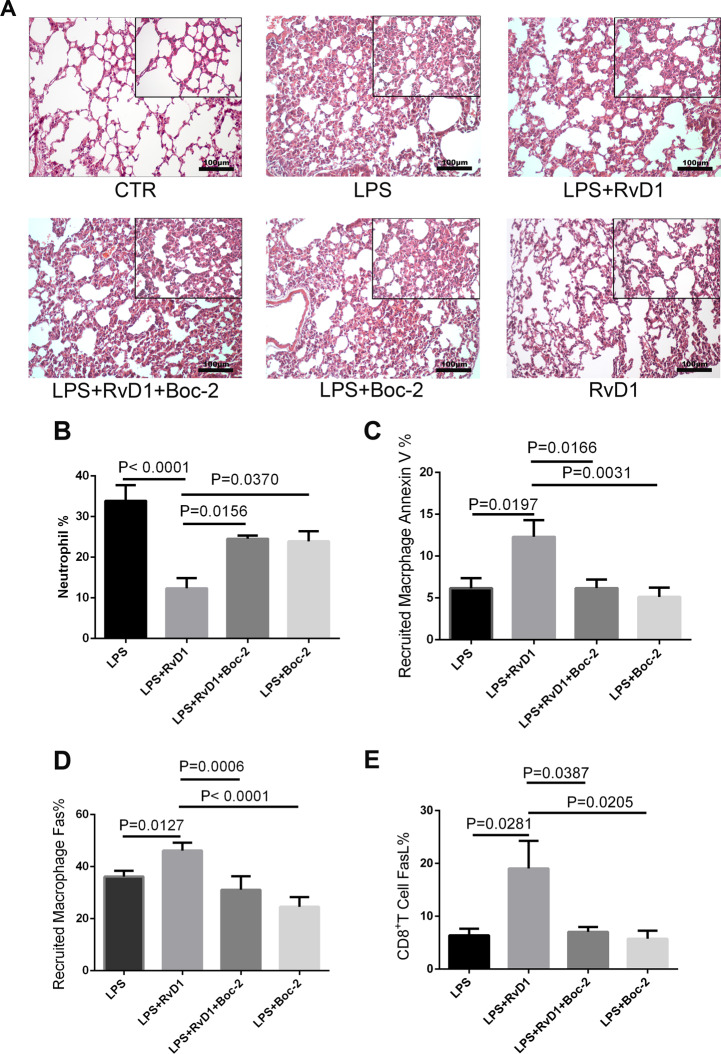
The protective effect of RvD1 on lung tissues is dependent on ALX/PRP2. **A** The pro-resolution effect of RvD1 on lung injury was abrogated by the ALX/PRP inhibitor. **B** Percentage of neutrophils in BALF. **C** Percentage of annexin V-positive (apoptotic) recruited macrophages. **D** Percentage of Fas on recruited macrophages and **E** FasL expression on CD8^+^ T cells were measured using flow cytometry.

The ALX/PRP2 inhibitor, BOC-2, was administered 1 h before the RvD1 injection. As seen in Fig. 7A, the protective effect of RvD1 on lung tissues was abolished by BOC-2. RvD1-induced neutrophil infiltration was abrogated by BOC-2 (Fig. 7B), whereas RvD1-induced apoptosis of the recruited macrophages (Fig. 7C), Fas expression (Fig. 7D) of the recruited macrophages, and FasL expression on CD8^+^ T cells (Fig. 7E) was abrogated by BOC-2.

## Discussion

ARDS is a common cause of respiratory failure in critically ill patients. Patients with ARDS who need intensive care have a mortality rate of 30–40% [20]. ARDS is characterized by the infiltration of excessive inflammatory cells. Thus, the removal of these cells could resolve inflammation. In our study, we found that neutrophils in the alveolar lavage fluid markedly increased in the first three days, subsequently decreased slowly, and then entered the phase of inflammation resolution. We found that treatment with RvD1 during the peak of inflammation restored the body weights of the mice, improved the pathophysiological changes in their lungs, decreased TNF-α, IL-1β, and IL-6 levels in the lung tissue homogenate, and reduced neutrophil counts in the BALF. However, it was worth noting that normal mice were unaffected. Together, these results indicated that RvD1 could protect the lung tissues of mice with ARDS during the resolution phase of inflammation.

A large number of neutrophils and recruited macrophages enter and accumulate in the lung after lung injury. The persistence of macrophages is a sign of chronic inflammation and can sometimes lead to fibrosis [16, 17]. Our findings suggested that treatment with RvD1 reduced the number of recruited macrophages, inhibited the expression of the recruited macrophages with the M1 phenotype, upregulated M2 expression, and promoted phagocytosis in vivo. In our in vitro study, we found that the expression of iNOS and CD86 after LPS stimulation was time dependent; it peaked at 12 h and then declined. Therefore, we detected the effect of RvD1 on BMDMs at 12 h. RvD1 suppressed the expression of iNOS and CD86 and promoted phagocytosis. Consistent with our results, a previous study has reported that RvD1 induces the polarization of macrophages and enhances phagocytosis [21, 22]. Our findings indicated that RvD1 could downregulate the recruited macrophage M1 phenotype, promote phagocytosis, and accelerate their removal to promote the resolution of inflammation. It is interesting to assess the presence and function of the recruited macrophages during the resolution phase of inflammation. Some recruited macrophages may migrate from tissues through the lymph and are capable of exiting the tissues to recirculate. We found that RvD1 increased the numbers of the recruited macrophages in the lymph nodes, suggesting that RvD1 could accelerate the removal of some of the recruited macrophages through the lymph. Previous studies show that inflammatory macrophages in the lungs, muscles, and nervous system are eliminated by apoptosis [18, 23, 24]. Two apoptotic pathways, namely the intrinsic and the extrinsic

pathways, have been identified in mammalian systems [25]. In the intrinsic apoptotic pathway, anti-apoptotic Bcl-2, Mcl-1, or pro-apoptotic Bax regulates mitochondrial membrane permeability [26]. In the extrinsic apoptotic pathway, the FasL on T cells binds to the Fas receptors on the targeted cells to accelerate apoptosis.

We found that treatment with RvD1 not only increased the level of Fas receptors and Fas mRNA on the recruited macrophages but also promoted the expression of FasL in CD8^+^ T cells in the resolution phase. The increased apoptosis of recruited macrophages was abrogated by treatment with Fas-blocking antibody. Treatment with RvD1 reduced Mcl-1 expression in the recruited macrophages; however, RvD1 administration did not change the expression of Bax in vivo. We also found that the apoptosis of BMDMs peaked at 6–12 h after stimulation with LPS (1 μg/mL). BMDMs were co-cultured with LPS and RvD1 for 12 h, and RvD1 was found to upregulate the expression of Fas receptors, but not Mcl-1, Bcl-2, or Bax. Taken together, these results suggest that RvD1 promoted apoptosis of the recruited macrophages mainly via the FasL-Fas pathway.

Caspases are a group of evolutionarily conserved cysteine proteins, functionally subdivided into initiators (caspase 8) and effectors (caspase-3) [27]. Fas signaling results in the formation of a death-inducing signal complex, which leads to the Fas-associated recruitment of caspase-8 through the adaptor protein of the death domain (FADD). Dimerization of caspase-8 triggers activation of the apoptotic cascade activation effector, caspase-3 [28]. The DNA repair enzyme poly (ADP-ribose) polymerase (PARP), also called poly (ADP-ribose) synthetase or poly (ADP-ribose) transferase (PADRT), is a ribozyme activated by the DNA chain for participation in DNA repair. PARP cleavage by caspase-3 is the defining feature of apoptosis [29]. Some special pro-inflammatory mediators, such as RvE1, have been shown to increase the activity of caspase-8 and caspase-3, and promote phagocytosis-induced apoptosis [30]. We found that RvD1 could increase the activity of caspase-8, upregulate the levels of cleaved caspase-3 and cleaved PARP, which indicated that this compound could promote apoptosis of the recruited macrophages partly through the FasL-Fas/caspase-3 signaling pathway.

ALX was the first receptor being identified. We found that the protective effect of RvD1 on lung tissues was abolished when the ALX inhibitor, BOC-2, was used. The RvD1-mediated reduction in neutrophil numbers was abrogated by BOC-2, and RvD1 treatment resulted in the upregulation of apoptosis. The expression of Fas and FasL on the recruited macrophages and CD8^+^ T cells, respectively, were also abolished by BOC-2, indicating that the response of RvD1 was ALX dependent.

**Fig. 8:**
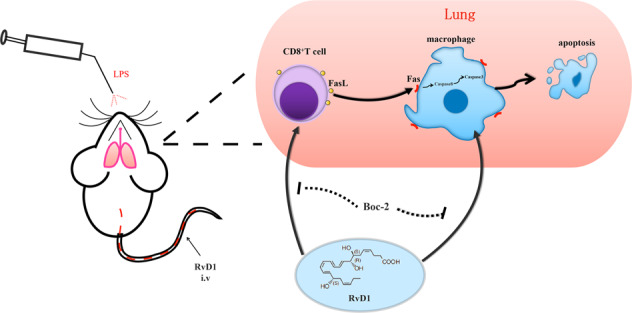
RvD1 accelerates the resolution of inflammation by promoting apoptosis of the recruited macrophages. RvD1 promoted apoptosis of the recruited macrophages in the resolution phase partly via the ALX/FasL-FasR/caspase-3 signaling pathway.

To summarize, our findings indicated that RvD1 enhanced efferocytosis and M2 phenotype in the recruited macrophages and reduced the inflammatory cytokines to alleviate LPS-induced tissue damage in the resolution phase. RvD1 also accelerated the removal of the recruited macrophages in the resolution phase mainly by apoptosis. Moreover, RvD1 promoted apoptosis of the recruited macrophages partly via the ALX/FasL-FasR/caspase-3 signaling pathway (Fig. 8). Our findings reinforce the concept of therapeutic targeting of apoptosis in the recruited macrophages and suggest that RvD1 may provide a new therapy in the management of ALI/ARDS.

## Materials and methods

### Reagents

LPS (*Escherichia coli* serotype 055:B5) was obtained from Sigma (St. Louis, MO). RvD1 was obtained from Cayman Chemical Company (Ann Arbor, MI). F4/80-PE-Cyanine7, Ly-6G-FITC, CD206-PE, CD86-PE, CD3-APC, and CD8-PerCy5.5 antibodies were procured from Invitrogen (Carlsbad, CA). FasL-PE and Fas-FITC were purchased from Biolegend (San Diego, CA) and the annexin V-PE/7-AAD apoptosis kit was purchased from MULTI SCIENCES. TNF-α, IL-1β, and IL-6 ELISA kits were obtained from R&D Systems (Minneapolis, MN). Rabbit antimouse iNOS antibody, anti-Bax (E63), anti-caspase-3, anti-Bcl-2, and mouse antimouse β-actin were procured from Abcam (Cambridge, MA). The Fas-blocking antibody, antimouse CD178 monoclonal antibody (clone MFL3) was procured from eBioscience (Rockford, IL).

### Animal studies

Specific pathogen-free adult C57BL/6 mice (female and male), each weighing 20–25 g, were obtained from Charles River Laboratories. The mice were housed under controlled temperature and humidity conditions on a day-night cycle and were provided access to food and water *ad libitum*. All procedures were conducted in accordance with the Guide for the Care and Use of Laboratory Animals. This study was approved by the Animal Studies Ethics Committee of Wenzhou Medical University.

Rats were randomized into each group. Atomization inhalation of LPS (1 mg/kg) was used to establish the lung injury mouse model. In the RvD1 group, the mice were administered RvD1 (100 ng/mouse) intravenously via the tail vein. In the LPS + RvD1 group, the mice were injected RvD1 intravenously via the tail vein 3 days after LPS stimulation, and the bronchoalveolar lavage fluid (BALF) was collected on day 5. For the Fas-blocking experiments, the Fas-blocking antibody (30 mg in 50 ml PBS) was administered intratracheally on days 3 and 4 after LPS, BALF was collected on day 5.

All the mice were included. For each group used in experiments, at least six mice were guaranteed for adequate power to detect a pre-specified effect size. Animal studies were blinded during the group allocation, experiments, and when assessing the outcomes.

### ELISA

TNF-α, IL-1β, and IL-6 levels in the lung tissue homogenate were measured using the respective ELISA kits according to manufacturers’ protocol.

### Cell culture

Bone marrow-derived macrophages (BMDMs) were isolated from 6-week-old BALB/C mice and their concentration was adjusted to 2 × 10^6^ cells/mL using RPMI1640 medium. The cells were plated in a 10-cm culture dish and were allowed to differentiate in a humidified incubator in an atmosphere of 5% CO_2_ at 37 °C. The medium was changed every 2–3 days. After 7 days, BMDMs (1 × 10^6^ cells/mL) were plated in 6-well plates. Cells were divided into four groups, namely, control, LPS, LPS + RvD1, and RvD1. All cells were cultured with LPS (1 μg/mL) in the presence or absence of RvD1 (100 nM) for 12 h, and efferocytosis and apoptosis were measured.

### Flow cytometry

BALF cells were collected and immediately treated with antimouse CD16/32 antibody (BD) for 20 min to block nonspecific binding. Macrophages were incubated with monoclonal antibodies directed at F4/80 (PE-Cy7), Ly6C (APC), and Fas (FITC). T lymphocytes were stained with a monoclonal antibody directed at CD3 (APC), CD4 (FITC), CD8 (PerCy5.5), FasL-PE, and analyzed using flow cytometry.

### Quantitative real-time RT-PCR

Recruited macrophages from the BALF were sorted using a CytoFLEX flow cytometer (Beckman Coulter). The total RNA of the recruited macrophages was isolated using a miRNeasy Mini Kit (Qiagen) according to the manufacturer’s protocol. The cDNA was synthesized using a reverse transcription kit (Thermo). Gene expression was detected using TB Green Premix Ex Taq < (Takara). Samples were normalized to the mRNA expression of the housekeeping gene (β-actin to analyze samples from mice) and the results are expressed as fold increase using the 2^−ΔΔCt^ method. The primer pairs of each gene were as follows:

Fas:5′-GCGGGTTCGTGAAACTGATAA-3′,5′-GCAAAATGGGCCTCCTTGATA-3′; FasL:5′-GGAGTGGTCCTTAATGCCT-3′,5′-TCTTTCTCTGTGCCTCTGC-3′;

MCL-1:5′-GACGAAACGGGAACTGGCTTGTC-3′, 5′-GCCGCGTTGTAGGTCGTGTAC-3′; BAX:5′-AGACAGGGGCCTTTTTGCTAC-3′, 5′-AATTCGCCGGAGACACTCG-3′; β-actin: 5′-GGCTGTATTCCCCTCCATCG-3′,5′-CCAGTTGGTAACAATGCCATGT-3′.

### Western blotting

Cell extracts were prepared by suspending the cells directly in the RIPA lysis buffer and PMSF for 15 min on ice, followed by centrifugation for 25 min at 12,000 × *g*. The supernatant was collected and protein concentration was measured using a protein assay kit. Protein samples (30 μg) were separated using SDS-PAGE and transferred to nitrocellulose membranes. Nonspecific protein binding was blocked using 10% non-fat dry milk-PBST buffer for 1 h at room temperature. The blots were then incubated overnight with the primary antibodies against iNOS, Bax, and Bcl-2 at 1:1000 dilutions. Then, they were washed three times with PBST and incubated for 1 h with the appropriate secondary antibodies coupled to horseradish peroxidase at 1:2000 dilutions. The protein bands were quantified using a UVP gel imaging system.

### Phagocytosis assay

To analyze the percentage of macrophages phagocytosis in vivo, FITC-LPS was administered via tracheal inhalation and RvD1 was injected through the tail vein. Twelve hours later, the percentage of FITC-LPS in the recruited macrophages was detected using flow cytometry. For in vitro experiments, BMDMs were cultured with FITC-LPS in the presence or absence of RvD1 for 12 h, and phagocytosis was determined using fluorescence microscopy.

### Statistical analysis

Data are presented as the mean ± SEM, and numbers of experiments (*n*) are indicated. Statistical tests are justified as appropriate for every figure, and the data meet the assumptions of the tests, including normal distribution. The estimated variance is similar between the groups that are being statistically compared. Data were analyzed using one-way ANOVA, Tukey test was used for posthoc comparison. A value of *p* < 0.05 was considered statistically significant. Statistical analyses were performed using SPSS 19.0 and Prism 6.0.

## Data Availability

The datasets used and/or analyzed during the current study are available from the corresponding author on reasonable request.
